# An Automated Digital Microfluidic System Based on Inkjet Printing

**DOI:** 10.3390/mi15111285

**Published:** 2024-10-23

**Authors:** Wansheng Hu, Ming Cao, Lingni Liao, Yuanhong Liao, Yuhan He, Mengxiao Ma, Simao Wang, Yimin Guan

**Affiliations:** 1 School of Microelectronics, Shanghai University, Shanghai 201800, China; 22723730@shu.edu.cn; 2 Shanghai Aure Technology Limited Company, Shanghai 201800, China

**Keywords:** spheroids, inkjet printing, controllable size, uniform preparation

## Abstract

Cellular interactions, such as intercellular communication and signal transduction, can be enhanced within three-dimensional cell spheroids, contributing significantly to cellular viability and proliferation. This is crucial for advancements in cancer research, drug testing, and personalized medicine. The dimensions of the cell spheroids play a pivotal role in their functionality, affecting cell proliferation and differentiation, intercellular interactions, gene expression, protein synthesis, drug penetration, and metabolism. Consequently, different spheroid sizes may be required for various drug sensitivity experiments. However, conventional 3D cell spheroid cultures suffer from challenges such as size inconsistency, poor uniformity, and low throughput. To address these issues, we have developed an automated, intelligent system based on inkjet printing. This system allows for precise control of droplet volume by adjusting algorithms, thereby enabling the formation of spheroids of varying sizes. For spheroids of a single size, the printing pattern can be modified to achieve a coefficient of variation within 10% through a bidirectional compensation method. Furthermore, the system is equipped with an automatic pipetting module, which facilitates the high-throughput preparation of cell spheroids. We have implemented a 3 × 3 spheroid array in a 24-well plate, printing a total of 216 spheroids in just 11 min. Last, we attempted to print mouse small intestinal organoids and cultured them for 7 days, followed by immunofluorescent staining experiments. The results indicate that our equipment is capable of supporting the culture of organoids, which is of great significance for high-throughput drug screening and personalized medicine.

## 1. Introduction

In traditional two-dimensional cell culture techniques, cells are typically cultured on flat surfaces, which, while simplifying experimental procedures, does not accurately replicate the complex in vitro cellular environment and interactions [1]. In recent years, three-dimensional (3D) cell culture techniques have gained widespread attention as an innovative method for simulating the natural in vivo environment within a three-dimensional space. Unlike two-dimensional cultures, three-dimensional cultures allow cells to grow and interact in a microenvironment that more closely resembles physiological conditions, thereby providing more authentic and comprehensive biological data [2]. Three-dimensional culture techniques have enhanced researchers’ understanding of cellular behavior, drug responses, and disease mechanisms and have shown broad application prospects in tissue engineering, drug screening, cancer research, and precision medicine [3,4].

Traditional 3D culture methods include entrapping, magnetized cell culture, and ultra-low adhesion techniques [5,6], which have to some extent advanced the development of 3D culture but also face challenges. These methods often encounter difficulties in controlling spheroid size, poor uniformity, low throughput, and complex operations in practical applications [7]. Entrapping methods may result in non-uniform spheroid sizes, magnetized cell culture may be limited by the intensity of the magnetic field and uniformity of size [8], and ultra-low adhesion techniques may have issues with unstable interactions between the culture material and cells, as well as higher costs. These limitations restrict the application and development of 3D culture in a wider range of fields. Therefore, there is an urgent need for new technologies and methods to optimize the generation process of 3D cell spheroids, improve their size control, uniformity, and ease of operation, and better support cellular biology research and applications [9].

**Figure 1:**
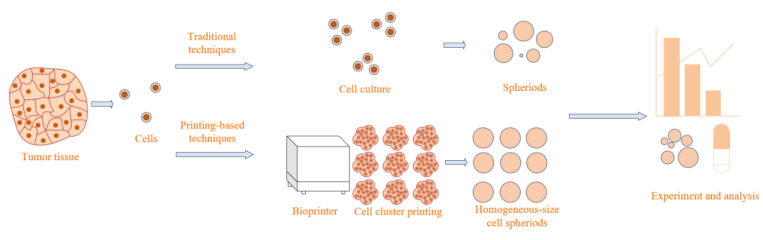
Flowchart of traditional cell sphere culture and bioprinting culture.

This paper addresses the time-consuming nature, size control challenges, and low throughput of various 3D cell spheroid preparation methods by designing an automated system based on inkjet printing, which enables the high-throughput preparation of size-controllable cell spheroids. As shown in Figure 1, we illustrate the difference in the process of preparing cell spheroids using entrapping methods and our printing system. Compared to entrapping, our automated system can print cells within a very small range in a short period of time. Due to the aggregation properties of cells, after the addition of culture medium, the cells aggregate to form a dense spheroid from a loose aggregate [10]. Additionally, by adjusting the control algorithms, the uniformity of the cell spheroids can be improved, achieving a coefficient of variation of less than 10% for cells printed onto a matrix gel. Furthermore, through control algorithms, the movement parameters of the nozzle can be precisely adjusted to form cell spheroids of different sizes. This has significant implications for drug screening, precision medicine, and other medical fields [11,12].

## 2. Construction of Automated Printing System

### 2.1. Chip Structure

**Figure 2:**
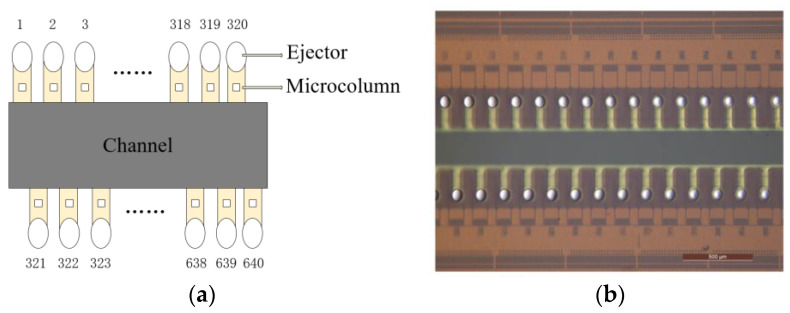
(**a**) A schematic diagram of the microfluidic chip. (**b**) A portion of the chip under the microscope; the scale is 500 μm.

The core component of this device is an integrated smart microfluidic chip designed and manufactured based on CMOS-MEMS technology, which integrates integrated circuits and microelectromechanical systems (MEMSs) together to achieve signal perception, signal processing, liquid drive integration, miniaturization, and high throughput. The chip is mainly composed of channels, filters, and ejectors. The channels are used to add and store biological ink and can control the movement of the ink through fluid backpressure. The filter uses a microcolumn array structure, which can effectively filter a large number of impurities. As shown in Figure 2a, the chip integrates 640 ejectors, with 320 ejectors distributed in two columns alternately along the channel, and the high-throughput nozzle design can effectively avoid experimental failure caused by the blockage of individual nozzles. Figure 2b presents an image of a portion of the chip under a microscope with a scale of 500 μm. Compared to traditional microfluidic channel-based chips, each ejector on this chip has an independent thermal drive circuit, and through the host computer software (version 3.0), it can send decoding data to the printing chip, controlling the opening and closing of each thermal drive circuit. After the experimenter adds biological ink to the channel, the ink will flow through the channel to the filter. After filtration, the ink reaches the ejector. When the ejector is working, the resistance in the thermal drive circuit will be powered to heat, and the ink droplet will be ejected from the ejector in the form of heat foaming. Each ejector ejects approximately 12 pL of droplets per operation, and the chip can achieve a sampling volume of 0 to 3840 pL through one movement; thus, the microfluidic chip has the advantages of microsample and high-precision cell manipulation, enabling the formation of high-throughput and standardized 3D in vitro models.

### 2.2. Construction of Printing System

**Figure 3:**
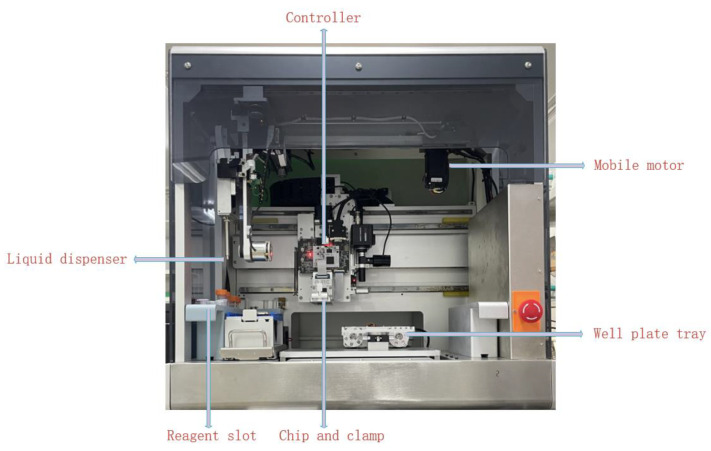
Bioprinting system.

As shown in Figure 3, the printing system is primarily composed of a well plate tray, a controller, a mobile motor, a liquid dispenser, a reagent slot, a chip, and a clamp. The system utilizes advanced droplet control technology for cell printing, achieving single-cell control within droplets. Before printing commences, researchers must first place the well plate into the tray and introduce the reagents into their corresponding slots. Once these preparations are complete, researchers configure specific printing arrays, initial printing positions, nozzle parameters, and ink settling times through the host computer software. After these configuration parameters are set, the main computer software sends instructions to the controller. Upon receiving instructions, the controller commands the movement of the mobile motors to drive the printing chip and liquid dispenser. When the printing chip reaches the configured position, the controller issues a printing command to the chip. At this point, the actuators on the printing chip activate the corresponding thermally driven circuits, causing the bioink to be ejected in the form of thermally induced bubbles to the target location, thereby achieving the printing operation.

This bioprinter uses advanced droplet control technology for fine cell printing by inoculating an exact number of cells onto a suitable extracellular matrix surface (such as Matrigel) [13,14], forming cell cluster arrays. After a certain period of culture, these cell clusters can grow into standardized cell spheroids on the extracellular matrix surface. Additionally, the printing system can adjust the size of the printed cell clusters and the number of arrays according to requirements, allowing control over the size of the formed three-dimensional cell spheroids and the number of microspheres.

## 3. Experimental Validation

### 3.1. Feasibility of Sphere Formation Verification

In this experiment, CHO cells were used as the experimental object to verify the feasibility of cell cluster formation after printing. The initial concentration of the CHO cell solution was 5 × 10^6^ cells/mL, with PBS as the solvent. Meanwhile, in the suspension culture system, we set up a CHO cell solution with the same initial concentration as the control group. Before printing, we placed a well-prepared matrix gel 24-well plate in the well plate slot and configured the printing parameters on the host computer software. The software will send the printing parameters to the moving control module. At this time, the pipette will first clean the channel. After the cleaning is complete, the pipette will add the CHO cell solution to the channel, waiting for the cells to settle. Once the cells have settled, the printing head can perform the printing operation. The printing head will print cells in a very small range, forming cell clusters. During the printing process, operations should be conducted in a sterile environment, and a humidifier should be prepared to keep the environmental humidity above 70% to prevent cell death due to the matrix gel drying out, allowing subsequent culture. After printing, the plate was placed in a 37 °C incubator for 40 min of incubation. After incubation, DMEM complete culture medium was added, and cell images were recorded on days 0, 1, 3, and 7.

**Figure 4:**
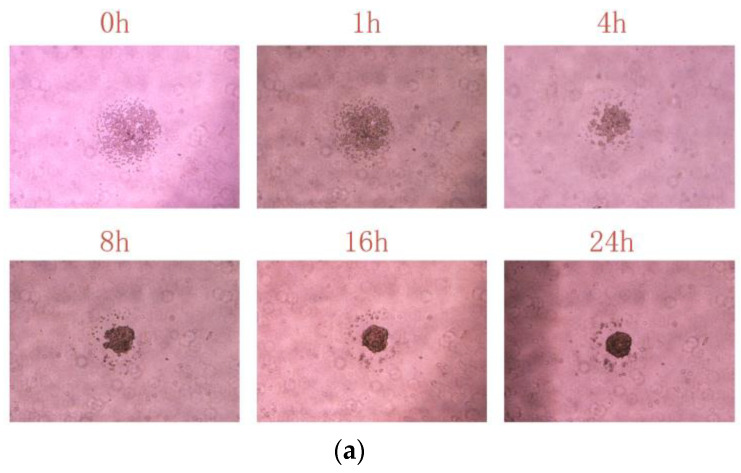

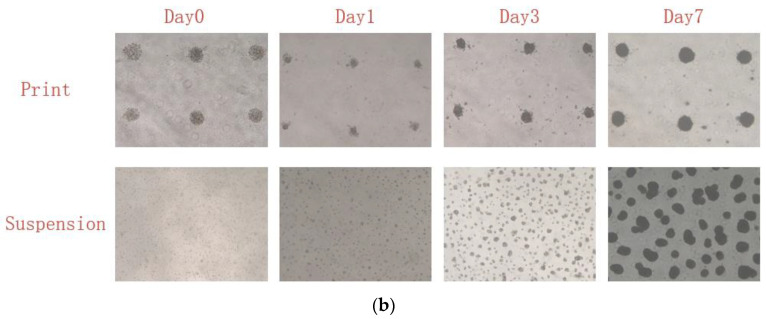
(**a**) Spontaneous aggregation process of printed cells within 24 h. (**b**) Comparative diameter of printing culture and suspension culture at days 0, 1, 3, and 7.

The bioprinting method involves seeding cells within an exceedingly small range, resulting in a higher initial cell density at the target location. Under appropriate cultivation conditions, these printed cells will spontaneously aggregate. Figure 4a shows the spontaneous aggregation process of printed cells within 24 h. As shown in Figure 4b, after 7 days of culture, the average size of the cell spheroids obtained using this method is 270 µm, whereas the average size of the cell spheroids produced by suspension culture is 134 µm. This implies that, to achieve spheroids of similar size, the clustered printing approach necessitates a shorter duration, leading to faster spheroid formation. Moreover, the cell spheroids cultured by clustered printing at a fixed location facilitate the manipulation of spheroids by experimental personnel for tasks such as drug susceptibility testing or other experiments.

The coefficient of variation (*CV*) is a dimensionless statistical measure used to indicate the relative dispersion of data [15]. It is calculated as shown in Equation (1) below, where *σ* represents the standard deviation and *μ* represents the mean.

(1) CV=σ/μ×100%

**Figure 5:**
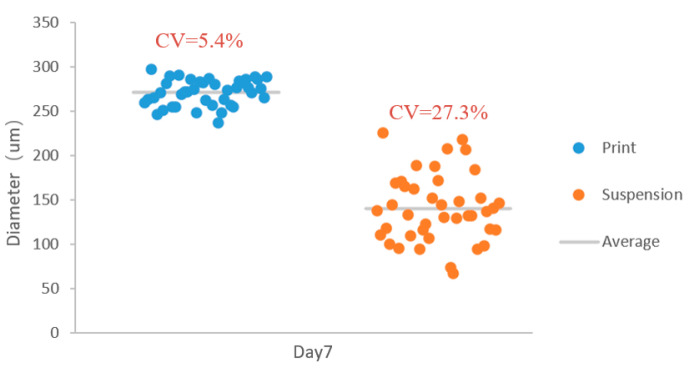
Comparison of diameter and coefficient of variation (*CV)* between printing culture and suspension culture at day 7.

In the context of cell spheroid culture, the *CV* value serves as an assessment of the uniformity of spheroid size and relative dispersal. A lower *CV* value indicates better uniformity of the spheroids, leading to higher stability and repeatability of experimental results. Figure 5 compares the *CV* values of the two culture methods at day 7 post-culturing, revealing that the *CV* value of the cell spheroids produced by printing culture is 5.4%, whereas that of the spheroids cultured by suspension is 27.3%. In the study by S Jiang et al. [16], the *CV* value of the diameter of the spheroids cultured using an automated printing platform at day 7 exceeded 10%, which is significantly higher than our observed 5.4%. This demonstrates that the printed culture yields spheroids of very similar sizes, enhancing the repeatability and reliability of experimental outcomes. This method is particularly beneficial for experiments requiring high uniformity in spheroid size and morphology, such as drug screening and stem cell differentiation research [17,18].

### 3.2. Uniformity Verification

For the homogenization validation, the printing system is capable of simultaneously fabricating multiple cell clusters to meet the demands of high-throughput preparation of spheroids. Taking a 24-well plate as an example, the printing system can support the printing of 12 cell clusters in a single movement for a 4 × 3 arrangement. Since the number of cells in the syringe is limited, the first ejection from the syringe will undoubtedly contain more cells than the second. With each syringe needing to eject 12 times, later ejections will have fewer cells. Consequently, if the printing operation is conducted with each syringe functioning identically, the number of cells in each cluster will decrease progressively.

To prevent this reduction in cell number, we modified the motion control algorithm of the control computer to allocate a certain proportion of syringes to each target location. The motion control algorithm enables the precise regulation of the thermal drive circuits for each ejector. By transmitting the number of ejectors required at the target location to the motion control module, the system activates the corresponding thermal drive circuits. Once these circuits are initiated, the respective ejectors perform printing operations at the designated location, thereby controlling the quantity of cells dispensed at that specific site.

**Figure 6:**
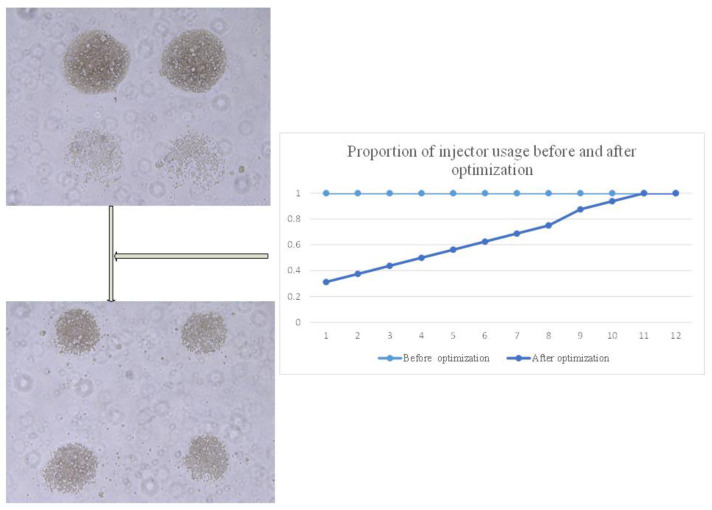
Print diagrams and nozzle usage ratios before and after optimization.

Furthermore, a bidirectional printing approach was adopted, where the printing head moves to the last target location and prints in reverse proportionally. As a result, as shown in Figure 6, the cell number at target location 1 is significantly higher than at target location 2 before the algorithm modification, but they become comparable after the modification.

### 3.3. Size Control Validation

In fields such as drug screening, metabolic research, and cancer research, it is often necessary to culture cell spheroids of different sizes [19]. The conventional approach involves using U-bottom ultra-low adhesion culture plates from Corning (Corning, NY, USA) for spheroid culture, with the effective means of controlling spheroid size being the alteration of the initial cell inoculation quantity. While this method is widely applied, it also has limitations and drawbacks. These include imprecise control of spheroid size, irregular spheroid shapes, difficulty in spheroid separation and analysis, poor experimental reproducibility, and high costs. These flaws are typically caused by factors such as the manufacturing process of the culture plates and the actions of the experimental personnel.

**Figure 7:**
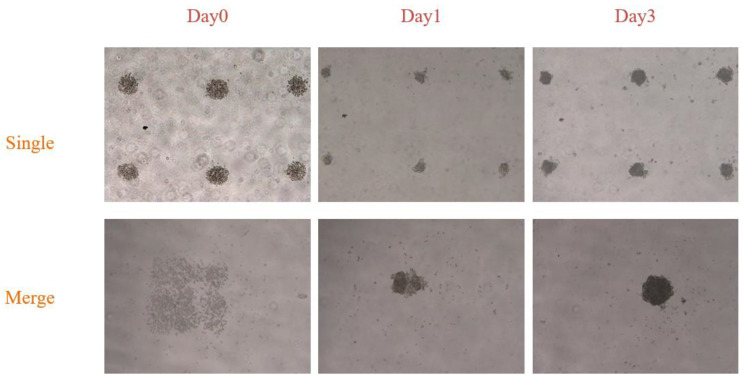
Culture diagrams of single and merge printing at day 0, day 1, and day 3.

To overcome these challenges, we modified the motion control algorithm to implement an adaptive function for the working ratio of the syringes. The experimental personnel can adjust the working ratio of the syringes based on the desired initial cell count. Additionally, to further expand the upper limit of spheroid size, we developed a new type of motion pattern that prints multiple cell clusters together. Stacking cells in the *Z*-axis direction can lead to issues such as cell scattering and difficulty in aggregating the cell clusters into a single spheroid after the culture medium is added. To address this phenomenon, we conducted printing experiments using merge distances ranging from 0 to 500 µm in increments of 10 µm. We found that a merge distance of 250 µm yielded the best results for spheroid culture in the later stages. As shown in Figure 7, printing with a 250 µm merge distance allowed the cell spheroids to reach a size of 402.61 µm by day 3, whereas the average size of spheroids cultured using single-point printing reached only 210.82 µm in the same period. This further increases the size of cell spheroids cultured within the same period.

### 3.4. Feasibility Verification of Organoid Printing

**Figure 8:**
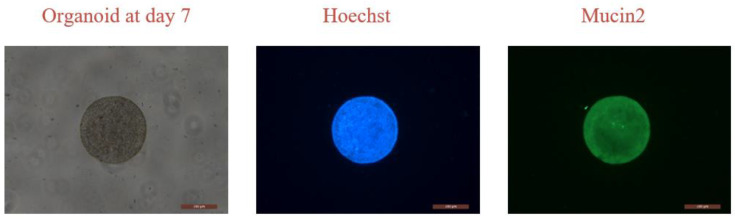
Intestinal organoid culture at day 7 and immunofluorescence stained with Hoechst and Mucin2.

Organoids require growth within a matrix gel system, and the popular method for this is entrapping. In some organoid research, different sizes of organoid spheres may be needed. However, entrapping methods struggle with controlling the size of organoid spheres and transferring them. Our printing method is compatible with the matrix gel system [20], and, thus, it is also applicable to organoids. We have already cultured organoids using our printing method, as shown in Figure 8, which depicts our experiment with mouse small intestinal organoids cultured for 7 days followed by immunofluorescent staining. The blue color represents Hoechst staining of cell nuclei, and the green color represents Mucin2 mucin staining [21]. We will subsequently attempt to culture more organoids and patient-derived organoids (PDOs) using this method.

## 4. Conclusions

This paper presents the development of an automated intelligent system based on inkjet printing, addressing the issues of size control, poor uniformity, and low throughput encountered in traditional 3D cell spheroid culture. The system, through precise algorithmic adjustments and control of the volume of ink ejected, is capable of forming cell spheroids of varying sizes to meet diverse experimental requirements, particularly in drug sensitivity testing, cancer research, and personalized medicine. Compared to traditional methods, an important improvement of this system is its high-precision size control, with the coefficient of variation for cell spheroids maintained within 10%, significantly enhancing the consistency and reliability of the experiments. Additionally, the system is equipped with an automatic pipetting module, further increasing the throughput of cell spheroid preparation. For instance, on a 24-well plate, it can print 216 cell spheroids in just 11 min, greatly improving the efficiency of the experiments. This high-throughput preparation method offers significant convenience for research that requires a large number of samples, such as drug screening, and significantly accelerates the experimental process. Furthermore, the intelligent design of the system ensures automated and stable operations, saving researchers a considerable amount of time and effort.
